# Mobile-Based Lifestyle Intervention in Women with Glucose Intolerance after Gestational Diabetes Mellitus (MELINDA), A Multicenter Randomized Controlled Trial: Methodology and Design

**DOI:** 10.3390/jcm9082635

**Published:** 2020-08-13

**Authors:** Caro Minschart, Toon Maes, Christophe De Block, Inge Van Pottelbergh, Nele Myngheer, Pascale Abrams, Wouter Vinck, Liesbeth Leuridan, Chantal Mathieu, Jaak Billen, Christophe Matthys, Babs Weyn, Annouschka Laenen, Annick Bogaerts, Katrien Benhalima

**Affiliations:** 1 Clinical and Experimental Endocrinology, Department of Chronic Diseases and Metabolism, KU Leuven, 3000 Leuven, Belgium; chantal.mathieu@uzleuven.be (C.M.); christophe.matthys@uzleuven.be (C.M.); katrien.benhalima@uzleuven.be (K.B.); 2 Department of Endocrinology, Imelda Hospital, 2820 Bonheiden, Belgium; Toon.Maes@imelda.be; 3 Department of Endocrinology-Diabetology-Metabolism, Antwerp University Hospital, 2650 Edegem, Belgium; Christophe.DeBlock@uza.be; 4 Department of Endocrinology, OLV Hospital Aalst, 9300 Aalst, Belgium; Inge.Van.Pottelbergh@olvz-aalst.be; 5 Department of Endocrinology, General Hospital Groeninge, 8510 Kortrijk, Belgium; nele.myngheer@azgroeninge.be; 6 Department of Endocrinology, GZA Hospital Sint-Vincentius, 2018 Antwerp, Belgium; Pascale.Abrams@gza.be; 7 Department of Endocrinology, GZA Hospital Sint-Augustinus, 2610 Wilrijk, Belgium; wouter.vinck@gza.be; 8 Department of Endocrinology, General Hospital Klina, 2930 Brasschaat, Belgium; Liesbeth.Leuridan@klina.be; 9 Department of Endocrinology, University Hospitals Leuven, 3000 Leuven, Belgium; 10 Department of Laboratory Medicine, University Hospitals Leuven, 3000 Leuven, Belgium; jaak.billen@uzleuven.be; 11 Department of Electrical Engineering, Processing Speech and Images, KU Leuven, 3000 Leuven, Belgium; barbara.weyn@kuleuven.be; 12 Centre of Biostatics and Statistical Bioinformatics, KU Leuven, 3000 Leuven, Belgium; annouschka.laenen@kuleuven.be; 13 Department of Development and Regeneration, KU Leuven, 3000 Leuven, Belgium; annick.bogaerts@kuleuven.be; 14 Faculty of Medicine and Health Sciences, Centre for Research and Innovation in Care (CRIC), University of Antwerp, 2610 Wilrijk, Belgium

**Keywords:** gestational diabetes mellitus, type 2 diabetes mellitus, postpartum, glucose intolerance, lifestyle intervention, mobile-based

## Abstract

The aims of the ‘Mobile-based lifestyle intervention in women with glucose intolerance after gestational diabetes mellitus (GDM)’ study (MELINDA) are: (1) to evaluate the prevalence and risk factors of glucose intolerance after a recent history of GDM; and (2) to evaluate the efficacy and feasibility of a telephone- and mobile-based lifestyle intervention in women with glucose intolerance after GDM. This is a Belgian multicenter randomized controlled trial (RCT) in seven hospitals with the aim of recruiting 236 women. Women in the intervention group will receive a blended program, based on one face-to-face education session and further follow-up through a mobile application and monthly telephone advice. Women in the control group will receive follow-up as in normal routine with referral to primary care. Participants will receive an oral glucose tolerance test (OGTT) one year after baseline. Primary endpoint is the frequency of weight goal achievement (≥5% weight loss if pre-pregnancy BMI ≥ 25 Kg/m^2^ or return to pre-gravid weight if BMI < 25 Kg/m^2^). At each visit blood samples are collected, anthropometric measurements are obtained, and self-administered questionnaires are completed. Recruitment began in May 2019.

## 1. Introduction

Gestational diabetes mellitus (GDM) is a frequent medical complication during pregnancy and is defined as diabetes diagnosed in the second or third trimester of pregnancy, provided that overt diabetes early in pregnancy has been excluded [1]. The incidence of GDM is rising globally and it represents an important modifiable risk factor for adverse pregnancy outcomes such as macrosomia and preeclampsia [2,3]. Shortly after delivery glucose values generally normalize, but the underlying beta-cell dysfunction often persists [4]. In the long term, women with a history of GDM have a seven-fold increased risk of developing type 2 diabetes mellitus (T2DM) compared to women without previous GDM [5,6]. About 50% of women with a history of GDM will develop T2DM within 10 years after delivery [5]. Moreover, women with a history of GDM progress more rapidly to T2DM compared to women with similarly elevated glucose levels [7]. Women with glucose intolerance (impaired fasting glucose (IFG) and/or impaired glucose tolerance (IGT)) in early postpartum are a particularly high risk group, with about 50% who will develop T2DM within five years after delivery [8].

Lifestyle modifications have been shown to be effective in the prevention or delay of T2DM when offered to high-risk middle-aged individuals. Two landmark trials, the United States Diabetes Prevention Program (DPP) and the Finnish Diabetes Prevention Study (DPS), showed a reduction of 58% in the incidence of T2DM in individuals presenting with IGT after an average of three years of intensive lifestyle interventions, with a weight loss of approximately 5 kg [9,10]. Subgroup analyses of the DPP trial, focusing on women with previous GDM, found a 53% reduction at the end of the trial and a 35% reduction after 10 years [7,11]. However, women in this trial were on average 12 years after index pregnancy, and the lifestyle intervention was very intensive and therefore challenging to implement in normal routine care, for both patient and clinic. In addition, there is limited evidence regarding the efficacy of lifestyle interventions in women with a recent history of GDM. Studies published in this population generally show limited benefits on metabolic outcomes, mostly because of low adherence rates and failure to produce meaningful behavior change due to barriers such as the need for child care, low family support and return to work [12,13,14]. A recent meta-analysis reviewed the benefits of lifestyle interventions for women with previous GDM and demonstrated moderate weight reductions, but only if the interventions were offered within the first six months after delivery [15]. The benefits shown were smaller than those observed in the large lifestyle intervention trials performed on older subjects.

Lifestyle interventions in women with previous GDM need to be adapted to address their unique barriers to behavior change in order to optimize adherence. A study of 300 women with a recent history of GDM showed that a telephone-delivered diabetes prevention program was associated with greater engagement compared to a group-delivered program (82% vs. 38%) in the postpartum period, and that the greater engagement was associated with greater reduction in weight and waist circumference [16]. Digital health interventions seem to be highly acceptable among postpartum women and should therefore be considered for lifestyle management in this population, provided that they focus on delivering behavior change strategies and addressing practical barriers faced by postpartum women [17]. Several mobile-based lifestyle interventions have shown potential for improved metabolic control in different populations, including women with a history of GDM and overweight or obese patients [18,19,20]. The B-slim project compared a conventional face-to-face weight loss and weight control program with a standalone mobile program and a combined program for overweight and obese patients [20]. They showed that the weight loss goal of 5% was reached in only 6% of the control group compared to 19% in the standalone mobile program, 28% in the conventional program and 46% in the combined program [20]. These results indicate that the content of a conventional weight loss program could be delivered through a combination of face-to-face coaching and a mobile health program without affecting the effectiveness of the intervention.

## 2. Objectives of the Melinda Study

Since women with glucose intolerance have a very high risk of developing T2DM, we designed the MELINDA study to investigate the efficacy and feasibility of a telephone- and mobile-based lifestyle intervention in women with glucose intolerance after a recent history of GDM. We also aim to evaluate the prevalence and risk factors of glucose intolerance in early postpartum in women with previous GDM.

Specific objectives are:

1. To evaluate whether a telephone- and mobile-based lifestyle coaching program leads to a higher frequency of weight goal achievement (≥5% weight loss if pre-pregnancy Body Mass Index (BMI) ≥ 25 Kg/m^2^ or return to pre-gravid weight if BMI < 25 Kg/m^2^).
2. To evaluate whether a telephone- and mobile-based lifestyle coaching intervention can reduce the incidence of metabolic syndrome, leading to an improved beta-cell function and lower insulin resistance in women with glucose intolerance after a recent history of GDM.
3. To investigate the prevalence of glucose intolerance and T2DM after a recent diagnosis of GDM based on the 2013 World Health Organization (WHO) criteria and to evaluate the risk factors of developing glucose intolerance postpartum.
4. To investigate factors related to success or failure in diabetes prevention and to develop materials and expertise to assist in the development of diabetes prevention programs in primary care.

## 3. Materials and Methods

### 3.1. Study Design and Setting

The MELINDA study is a multicenter randomized controlled trial with the participation of 7 hospitals to test the efficacy of a telephone- and mobile-based lifestyle intervention for one year to stimulate weight loss in women with glucose intolerance after a previous pregnancy with GDM. The Leuven University Hospital is the coordinating center.

In order to maintain uniform criteria for the diagnosis of GDM, all participating centers use the 75 g oral glucose tolerance test (OGTT) with the ‘International Association of Diabetes in Pregnancy Study Groups’ (IADPSG) criteria, now commonly referred to as the 2013 WHO criteria for GDM [21,22]. As part of normal routine, women with a history of GDM are offered a 75 g OGTT between 6 and 16 weeks after delivery. Data from eligible and consenting women are collected during the postpartum OGTT, including baseline characteristics and data on medical and obstetrical history. Women with glucose intolerance based on the postpartum OGTT are offered participation in the intervention randomized controlled trial (RCT). Participants are randomized with a 1:1 allocation ratio and stratified by center and baseline BMI to the telephone- and mobile-based lifestyle intervention or to the control group. A password-protected, computer-generated, variable block randomization is used to prevent disclosure of the allocation sequence to recruiters. This results in concealed and varying block sizes of two and four patients.

**Figure 1:**
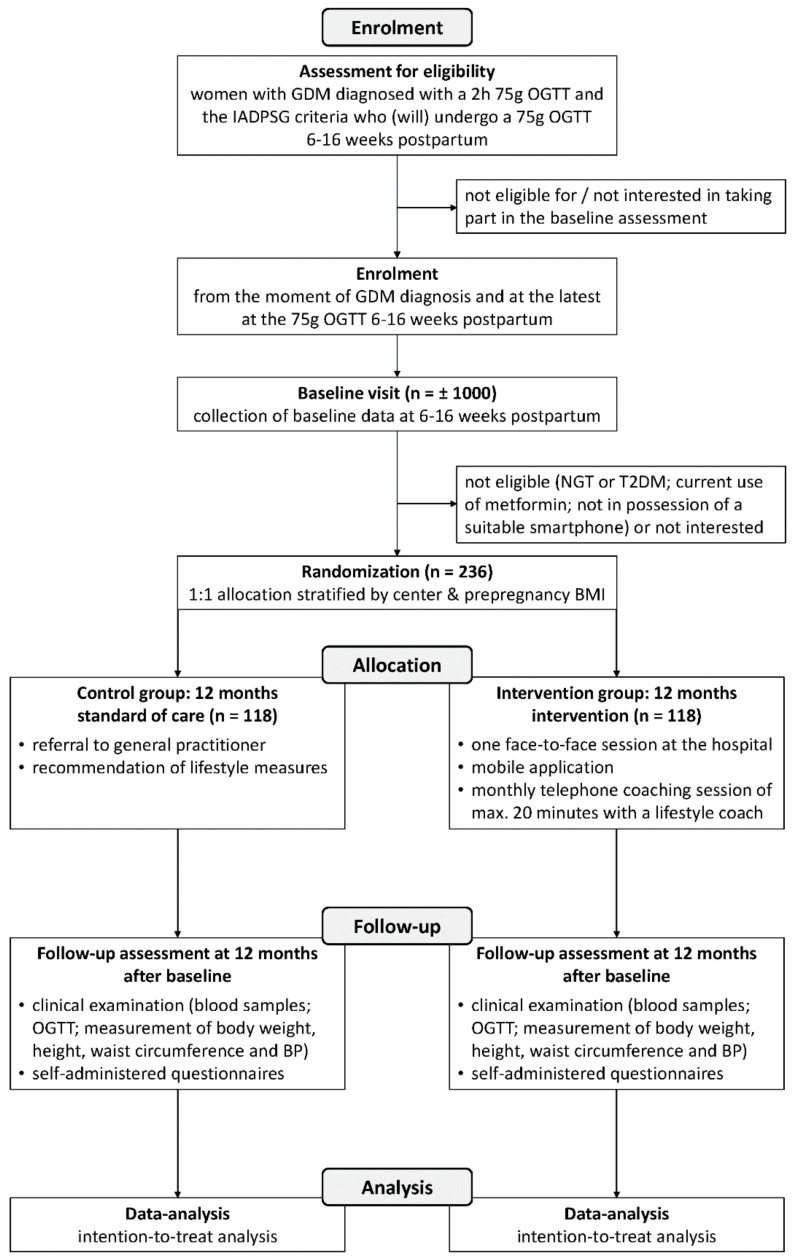
‘Standard Protocol Items: Recommendations for Interventional Trials’ (SPIRIT) flow diagram showing the flow of participants through the phases of the Mobile-based lifestyle intervention in women with glucose intolerance after gestational diabetes mellitus (MELINDA) randomized controlled trial. GDM: gestational diabetes mellitus; OGTT: oral glucose tolerance test; IADPSG: The International Association of Diabetes and Pregnancy Study Groups; NGT: normal glucose tolerance; T2DM: type 2 diabetes mellitus; BMI: Body Mass Index; BP: blood pressure.

Both women in the intervention and control group receive a 75 g OGTT one year after the postpartum OGTT as part of the trial. The ‘Standard Protocol Items: Recommendations for Interventional Trials’ (SPIRIT) flow diagram illustrates the design of the study (Figure 1). The study is registered in ClinicalTrials.gov as NCT03559621 and was approved by the Medical Ethical Committees of all participating centers (Belgian number: B322201837047).

### 3.2. Recruitment and Eligibility

The cohort is recruited from two university centers (Leuven University Hospital and Antwerp University Hospital) and six non-university centers (Imelda Hospital, Bonheiden, OLV Hospital, Aalst, General Hospital, Groeninge Kortrijk, GZA Hospital site, Sint-Augustinus, GZA Hospital site, Sint-Vincentius and General Hospital, Klina Brasschaat). All Dutch, English or French speaking women aged 18 or older with GDM based on the 2013 WHO criteria and presenting at the 75 g OGTT 6–16 weeks after delivery are invited to participate. A first written informed consent is obtained from the participants before any trial-related activities are performed. Women with glucose intolerance based on the 75 g OGTT 6 to 16 weeks after delivery are offered participation in the intervention RCT and a more extensive written informed consent is therefore obtained from this group. Glucose intolerance is defined as IFG [fasting plasma glucose (FPG) 100–125 mg/dL] and/or IGT (2-h glucose value on the OGTT between 140–199 mg/dL) as described by the American Diabetes Association (ADA) [1]. The maximum study duration for participants in the RCT is one year. The planned recruitment period is 2.5 years.

Exclusion criteria for the baseline study:

- current use of medication that can affect glucose values (such as glucocorticoids) or receiving treatment for glucose intolerance (such as metformin)
- women who do not undergo the 75 g OGTT 6–16 weeks at the hospital
- women who are not diagnosed with GDM based on the 2013 WHO criteria
- insufficient Dutch, English or French language skills

Additional exclusion criteria for the RCT:

- diabetes (FPG ≥126 mg/dL and/or 2-h glucose value ≥ 200 mg/dL)
- normal glucose tolerance
- current use of metformin
- health limitations or treatments (as assessed by the local investigator according to a standardized protocol) which would restrict participation in the intervention trial
- not in possession of a suitable smartphone (iOS or Android)

### 3.3. Study Visits 

#### 3.3.1. Baseline Measurements during the 75 g OGTT 6–16 Weeks After Delivery

Baseline characteristics are collected for all eligible women through a clinical examination, self-administered questionnaires, collection of blood samples and extraction of data on medical history and pregnancy from the electronic medical records. A standard 75 g OGTT is performed with blood samples taken fasting and at 30, 60 and 120 min. Aliquots of serum and plasma are stored for future measurements.

#### 3.3.2. Enrolment in the Intervention RCT

Women with glucose intolerance are included in the RCT and randomly assigned to one of the two comparison groups with 1:1 allocation, stratified by center and pre-pregnancy BMI. Assignment is allocated automatically and received through a secure, password protected program to guarantee adequate concealment of allocation.

#### 3.3.3. Intervention Group

Women in the intervention group receives a blended lifestyle intervention for one year based on a unique combination of one face-to-face meeting, monthly telephone coaching and the use of a mobile application (MELINDA app) to promote healthy lifestyle behaviors. Within one month after the 75 g OGTT, participants receive an individual face-to-face coaching session at the hospital, given by a health professional trained in motivational interviewing. During this session, participants receives information on the long-term risks associated with GDM, the importance of a healthy lifestyle and how this can be achieved. In addition, information on the coaching program and instructions for using the MELINDA app are provided. After the face-to-face coaching session, participants receive a monthly telephone coaching session of maximum 20 min to allow for personalized feedback and support, focusing on individual needs. After one year, participants undergo a 75 g OGTT with the same examination as during the postpartum visit 6–16 weeks after delivery.

#### 3.3.4. Control Group

At the postpartum visit 6–16 weeks after delivery, women in the control group receive general information from the diabetes educator on the long-term risks associated with GDM and the need for a healthy lifestyle to prevent T2DM. Additionally, they are referred to primary care for further follow-up, in line with normal routine. As part of the study, after one year, participants undergo a 75 g OGTT with the same examination as during the postpartum visit 6–16 weeks after delivery. The Melinda app has not been made publicly available and was therefore not accessible to participants of the control group.

### 3.4. Key Aspects of the Blended Lifestyle Intervention

The lifestyle program for the MELINDA study has been developed by several research teams of the KU Leuven university. It builds on lifestyle interventions from previous studies by our research group [20,23,24] and is adapted to meet the particular needs of women with a recent history of GDM. This blended approach runs through a specific platform, which consists of three main functionalities: (1) a dashboard for the lifestyle coach; (2) the personalized MELINDA coaching app for the participants; and (3) integration of objective measures obtained via external devices (Xiaomi Mi Band pedometer connected to the MELINDA app).

The MELINDA app has been developed for the purpose of this study after consulting patients and healthcare professionals. The app and dashboard are developed by the Medical image computing department of the KU Leuven. The platform for the application is based on the existing IT framework from the B-Slim project [20]. In a first phase, a small feasibility study was conducted with eighty volunteers with a recent history of GDM to provide feedback on the functioning and usability of the MELINDA app. The app was tested during one month, evaluated through an online questionnaire and adapted according to the comments of the volunteers. After the app was adjusted, it was presented and made available to the lifestyle coaches in a training session. In this phase, some final adjustments were made to the content and functionality of the app, based on the comments of the coaches.

The app consists of a coaching module and a data entry module. The data entry module asks information about dietary intake, food literacy related determinants, weight, waist circumference and motivational status. The coaching module provides a 12-week dietary coaching trajectory interspersed with a 12-week physical activity coaching trajectory. A model of food literacy is used to focus on a broader set of processes, such as food planning, selecting the right foods, food preparation, eating habits and evaluating information about food [25]. The MELINDA app uses a set of limited questions on food literacy to produce tailored goals and tips. Physical activity is automatically monitored via a pedometer connected to the app in order to tailor the physical activity advice and skills training. If goals are achieved within six months, the app will help to maintain and support the current healthy lifestyle. If goals are not achieved, based on the telephone contact and the monitored data new goals and/or recommendations will be formulated in consultation with the participant and new tailored modules will be offered to optimize lifestyle.

During the monthly telephone coaching sessions, the lifestyle coach consults the dashboard that provides an overview of the evolution of weight, waist circumference, physical activity and motivational status of the participant. This information is used to optimize the advice and to zoom in on those areas with most needs and priorities.

### 3.5. Data Collection

An overview of all data collection procedures is provided in Table 1.

#### 3.5.1. Blood Collection

A fasting blood test is performed at 6–16 weeks after delivery for all baseline participants and after one year for the participants in the RCT.

At 6–16 weeks after delivery, the fasting blood test consists of HbA1c, lipid profile (total cholesterol, triglycerides, HDL and LDL cholesterol) and a 2-h 75 g OGTT with measurements of glucose and insulin fasting, at 30 min, 60 min and 120 min. For the 2-h 75 g OGTT, participants are instructed to fast for at least 10 h and not to smoke, engage in any physical activity, or give breastfeeding during the test. They are also instructed to drink only water, but no coffee, cola or any drink containing sugar or caffeine. All analyses, except for insulin, are performed locally at each participating center in line with normal routine. The insulin samples are analyzed centrally at the Leuven University Hospital (UZ Leuven).

For participants in the RCT, an additional 2-h 75 g OGTT with the same lab tests as baseline is performed one year. All laboratory tests are analyzed centrally at UZ Leuven to ensure uniformity. Only the analyses of glucose (fasting, 30 min, 60 min and 120 min) are performed locally so that there is no delay in diagnosing diabetes. Every three months, blood samples are collected for longer term storage in a -80 °C freezer at the biobank of UZ Leuven.

#### 3.5.2. Clinical Examinations 

Blood pressure (BP), height, weight and waist circumference are measured during the 75 g OGTT at baseline and after 1 year. BP is measured twice with a five min interval using an automated blood pressure monitor (Omron Philips large 34–44 cm). Height is measured to the nearest 0.5 cm using a calibrated wall-mounted stadiometer. Weight is measured using a calibrated portable Tanita HD 382 digital scale, which measures up to 150 Kg. BMI is calculated as Kg/m^2^. Waist circumference is measured in centimeters by applying the tape directly on the skin, horizontally at the lateral level that is midway between the iliac crest and the lowest lateral portion of the rib cage.

#### 3.5.3. Self-Administered Questionnaires

Questionnaire on general habits and socio-economic factors: a self-designed questionnaire was previously used in the Belgian Diabetes in Pregnancy Study (BEDIP-N) study [26] to extensively collect information on socio-economic status and habits.

Food Frequency Questionnaire (FFQ) validated for the Belgian population [27]: a questionnaire containing questions on frequency and portion size of consumption of foods and beverages.

International Physical Activity Questionnaire Long Form (IPAQ-LF) validated for use in the Belgian population [26,28]: questionnaire measuring different areas of physical activity such as job-related physical activity, transportation, house work and caring for family, recreation and time spent sitting. We added a question on time watching television or playing computer games to better assess sedentary behavior.

Center for Epidemiologic Studies-Depression (CES-D) questionnaire: widely used in pregnant and postpartum women to asses symptoms of clinical depression over the past seven days [29].

Questionnaire on breastfeeding and contraception: a self-designed questionnaire as previously used in the BEDIP-N study to extensively collect information on the duration and frequency of breastfeeding as well as on the type of contraception used [26].

36-Item Short Form Health Survey (SF-36): a set of generic, coherent, and easily administered quality-of-life measures that is validated for use in the maternity context [30]. Data from this questionnaire will be used to calculate Quality-Adjusted Life Years (QUALY’s) to explore the cost-effectiveness of the lifestyle intervention.

Risk Perception Survey For Developing Diabetes (RPS-DD): since it has been shown that women with a history of GDM often underestimate their risk of developing T2DM, a validated questionnaire to evaluate their perception of the development of diabetes is used [31].

Treatment Self-Regulation Questionnaire (TSRQ): a validated questionnaire that is widely used in the study of behavior change in healthcare settings to evaluate motivation for lifestyle change [32,33].

Spielberger State-Trait Anxiety Inventory (STAI-6) questionnaire: the validated short version STAI-6 is used to measure levels of anxiety state [34,35].

Sense of Coherence (SOC) questionnaire: a 13-item questionnaire to assess comprehensibility, manageability, and meaningfulness of one’s life [36].

General questionnaire on the acceptability and subjective quality of the MELINDA lifestyle intervention: a self-designed questionnaire evaluating the coaching system, the use and user-friendliness of the MELINDA app, and the monthly telephone coaching.

#### 3.5.4. Melinda App

The following data are collected for participants in the intervention group from the MELINDA app: self-reported weight and motivational status at least once a month; self-reported waist circumference at least once every three months; steps collected from the pedometer connected to the Melinda app; and app-based tracking to evaluate the use of the MELINDA app.

### 3.6. Outcomes of the Study

#### 3.6.1. Primary Outcome

The primary outcome of the RCT is the number of women reaching the weight-loss goal ≥5% of body weight when BMI ≥ 25 Kg/m^2^ or returning to pre-gravid weight if BMI < 25 Kg/m^2^ (based on pre-pregnancy BMI or, if not available, based on BMI in early pregnancy).

#### 3.6.2. Secondary Outcomes

- frequency of T2DM based on the ADA criteria after one year [37] and risk factors for the development of T2DM after one year
- frequency of glucose intolerance and the risk factors of glucose intolerance in early postpartum
- frequency of the metabolic syndrome based on the WHO criteria [38]
- insulin resistance and beta-cell function. The insulin sensitivity will be measured using the Matsuda index and the reciprocal of the homeostasis model assessment of insulin resistance (1/HOMA-IR) [39,40]. Beta-cell function will be assessed by HOMA-B, the insulinogenic index divided by HOMA-IR, and by the insulin secretion sensitivity index [41,42]. All these measures have been validated for use in women with GDM [26].
- mean weight loss
- duration of breastfeeding and rate of exclusive breastfeeding
- quality of life, symptoms of depression and anxiety
- motivation for behavior change and perceived risk of developing diabetes
- dietary quality
- intensity and duration of physical activity
- process outcome: the percentage of women adhering to the protocol of intervention, by monitoring the use of the MELINDA app and the number of telephone coaching sessions.

#### 3.6.3. Pregnancy and Delivery Outcome Data (Collected from the Electronical Medical Record)

Maternal data: parity; pre-pregnancy weight; gestational week of GDM diagnosis; results of the 50 g glucose challenge test (GCT) and 75 g OGTT during pregnancy; treatment of GDM during pregnancy; pregnancy complications such as preeclampsia (de novo BP ≥ 140/90 mmHg > 20 weeks with proteinuria or signs of end-organ dysfunction), eclampsia, gestational hypertension (de novo BP ≥ 140/90 mmHg > 20 weeks), preexisting hypertension and pregnancy-induced cholestasis.

Delivery data: pregnancy duration; type of labor (spontaneous, induced or caesarean before labor) and indications; type of delivery (spontaneous vaginal, forceps or vacuum, caesarean section during labor or planned caesarean section) and indications.

Neonatal data: macrosomia (>4 Kg), large for gestational age (LGA)(birth weight > 90 percentile according to standardized Flemish birth charts adjusted for sex of the baby and parity) [43], small for gestational age (SGA)(birth weight < 10 percentile according to standardized Flemish birth charts adjusted for sex of the baby and parity) [43], preterm delivery (<37 completed weeks), 10 min Apgar score, shoulder dystocia, birth trauma, neonatal respiratory distress syndrome, neonatal hypoglycemia (defined as glycemia < 40 mg/dL or need for intravenous dextrose), neonatal jaundice, duration and indication for admission on the neonatal intensive care unit (admission defined as >24 h). 

### 3.7. Power Calculation and Statistical Analysis

#### 3.7.1. Sample Size

Based on the results of the B-slim project, we assume that 20% of women in the control group will reach the weight-loss goal compared to 40% in the intervention group [20]. The sample size is calculated to show with 80% power and 5% significance level a difference in the proportion reaching the weight-loss goal after 1 year. The sample size calculation is based on a two-sided Chi-square test. Assuming a drop-out rate of 30%, a total sample size of 236 for the RCT is needed.

Data from our research group at UZ Leuven show that 44% of women with a recent history of GDM based on the two-step screening strategy and the 2013 WHO criteria have glucose intolerance three months after delivery [44]. Since UZ Leuven attends to a higher risk population than many other centers in Belgium, a lower overall rate of 30% of glucose intolerance in early postpartum across all participating centers is estimated. We further estimate that 30–50% of women with glucose intolerance postpartum will agree to participate in the trial. In order to enroll 236 women with glucose intolerance in the RCT, we estimate that about 1000 participants will have to be recruited baseline in early postpartum.

#### 3.7.2. Data Analysis

Descriptive statistics will be presented as frequencies and percentages for categorical variables and means with standard deviations or medians with interquartile range for continuous variables. Comparison of the outcomes in the intervention groups will be based on logistic regression analyses for binary outcomes, proportional odds models for ordinal data, or linear models for continuous outcomes. The principle of intention-to-treat will be adopted for all outcomes. Inferential analyses will be performed for the following secondary endpoints: frequency of normal glucose tolerance, frequency of the metabolic syndrome, insulin resistance and beta-cell function, weight loss and motivation for behavior change. The remaining secondary endpoints will be analyzed using descriptive statistics. The number of patients with missing data will be statistically compared across intervention arms to check for any imbalance. The method of multiple imputation will be adopted for all outcome variables, in case of evidence of such imbalance (*p* < 0.20). Logistic regression models will be used for binary outcomes, or linear models for continuous outcomes. Imputation models will include the study group and patient characteristics. 10 imputations will be performed. All analyses will be performed as two-sided tests at 5% significance level.

### 3.8. Quality Control Procedures

Every participating site is opened after a first initiation visit. Monitoring visits are conducted every four months to check adherence of the participating sites to the protocol and completeness of data collection forms. Each participant gets a subject identification number to ensure confidentiality of the data. All data collected in this study are referred to by subject identification number only. All obtained data are entered in the Good Clinical Practice (GCP) compliant Electronic Data Capture (EDC) platform ‘Castor EDC’ [45].

## 4. Discussion

Lifestyle modifications need to be adapted for women with previous GDM to address their unique barriers to behavior change in order to optimize adherence. Digital health interventions seem to be highly acceptable among postpartum women and should therefore be considered for lifestyle management in this population, provided that they focus on delivering behavior change strategies and addressing practical barriers faced by postpartum women. Mobile interventions should therefore be evaluated so that the intervention can be adapted to the time demands of young mothers, be less resource intensive and be more suited for implementation in primary care. A limitation in previous studies is the inclusion of women with normal glucose tolerance, which might explain the difficult engagement of participants since they might perceive themselves to be at low risk of diabetes.

To our knowledge, this is the first RCT to investigate the efficacy of a blended telephone- and mobile-based lifestyle intervention in women with glucose intolerance shortly after a pregnancy with GDM. The intervention consists of a novel combination of one face-to-face meeting, coaching by telephone and mobile support through the MELINDA app. Moreover, the coaching program is based on the new concepts of precision public health prevention and food literacy. The MELINDA app is made available in Dutch, French and English, so that ethnic minorities and non-native Dutch speaking women (often a higher risk group) can also participate. By collaboration with seven hospitals (both university and non-university centers), a multi-ethnic population can be recruited, representative for the background population with GDM in the Northern part of Belgium (Flanders). Additionally, this study will provide accurate data on the prevalence of glucose intolerance and T2DM in early postpartum across Flanders and will therefore allow the evaluation of the extent to which the newer 2013 WHO criteria for GDM will affect the demand for diabetes prevention services postpartum.

While this RCT has many strengths, it might also have some potential limitations. A potential limitation is that the study is not powered to detect a difference in T2DM risk as a primary outcome. However, our primary outcome—based on weight-loss goals—is a strong predictor of T2DM development, as different studies have shown that moderate weight loss is effective in reducing risk of T2DM [9,10,46]. Although we have taken as many measures as possible to include a broad population, some exclusion bias might occur unintentionally, especially from foreign-speaking immigrants or women who do not have the appropriate smartphone at their disposal.

In conclusion, lifestyle interventions in women with a recent history of GDM need to be adapted to address their unique barriers to behavior change. The MELINDA study investigates a novel telephone- and mobile-based lifestyle intervention to promote a healthy lifestyle in women with glucose intolerance after a recent history of GDM.

## Figures and Tables

**Table 1:** Data collection procedures in the MELINDA trial.

Outcomes	Assessments	Timing of Assessments						
		Baseline	1 Month *	Monthly * (Month 2 to 11)	3 Months *	6 Months *	9 Months *	12 Months **
**Outcomes Collected from Medical Records**								
Demographic data	Age; date of birth	x						
Medical and obstetric history	Pre-pregnancy weight and BMI; weight at delivery; parity; previous pregnancy outcomes	x						
**Patient-Reported Outcomes**								
General lifestyle behaviour and socio-economic factors	Self-constructed questionnaire	x						
Medication use	Questioned by study nurse	x	x	x	x	x	x	x
Diet	Food Frequency Questionnaire (FFQ)	x			x	x	x	x
Physical activity	International Physical Activity Questionnaire Long Form (IPAQ-LF)	x						x
Mental health and well-being	Center for Epidemiologic Studies -Depression (CES-D) questionnaire and Spielberger State-Trait Anxiety Inventory (STAI-6) questionnaire	x						x
Quality of life	36-Item Short Form Health Survey (SF-36)	x						x
Diabetes risk perception	Risk Perception Survey for Developing Diabetes (RPS-DD)	x						x
Motivation for lifestyle change	Treatment Self-Regulation Questionnaire (TSRQ)	x (v2)						x (v3)
Breastfeeding and anticonception	Self-constructed questionnaire	x						x
Sense of coherence	Sense of Coherence (SOC) questionnaire	x						x
Acceptability and subjective quality of the lifestyle intervention	Self-constructed questionnaire							x
**Clinical and Biochemical Outcomes**								
Anthropometry	Height (only baseline), weight, waist circumference, blood pressure, BMI	x						x
Glucose and insulin	Fasting 75 g OGTT (0, 30, 60 and 120 min)	x						x
HbA1c	Fasting	x						x
Lipid profile	Total cholesterol, triglycerides, HDL, LDL (fasting)	x						x
**Outcomes Collected from the MELINDA App**								
Weight	Self-reported weight in the MELINDA app			x				
Motivational status	Self-reported motivational status in the MELINDA app			x				
Waist circumference	Self-reported waist circumference in the MELINDA app				x	x	x	
Physical activity	Steps collected from pedometer connected to Melinda app							x
Use of the MELINDA app	App-based tracking to evaluate the use of the MELINDA app			x				x

* Only for participants in the intervention group of the randomized control trial (RCT); ** Only for participants in the intervention group and control group of the RCT.

## References

[1] American Diabetes Association (2017). Standards of Medical Care in Diabetes-2017. Diabetes Care.

[2] Crowther C.A., Hiller J.E., Moss J.R., Mcphee A.J., Jeffries W.S., Robinson J.S. (2005). Effect of Treatment of Gestational Diabetes Mellitus on Pregnancy Outcomes. N. Engl. J. Med..

[3] Landon M.B., Spong C.Y., Thom E., Carpenter M.W., Ramin S.M., Casey B., Wapner R.J., Varner M.W., Rouse D.J., Thorp J.M. (2009). A multicenter, randomized trial of treatment for mild gestational diabetes. N. Engl. J. Med..

[4] Buchanan T.A. (2001). Pancreatic B-Cell Defects in Gestational Diabetes: Implications for the Pathogenesis and Prevention of Type 2 Diabetes. J. Clin. Endocrinol. Metab..

[5] Bellamy L., Casas J.-P., Hingorani A.D., Williams D. (2009). Type 2 diabetes mellitus after gestational diabetes: A systematic review and meta-analysis. Lancet.

[6] Benhalima K., Lens K., Bosteels J., Chantal M. (2019). The Risk for Glucose Intolerance after Gestational Diabetes Mellitus since the Introduction of the IADPSG Criteria: A Systematic Review and Meta-Analysis. J. Clin. Med..

[7] Ratner R.E., Christophi C.A., Metzger B.E., Dabelea D., Bennett P.H., Pi-Sunyer X., Fowler S., Kahn S.E. (2008). The Diabetes Prevention Program Research Group. Prevention of Diabetes in Women with a History of Gestational Diabetes: Effects of Metformin and Lifestyle Interventions. J. Clin. Endocrinol. Metab..

[8] Gerstein H.C., Santaguida P., Raina P., Morrison K.M., Balion C., Hunt D., Yazdi H., Booker L. (2007). Annual incidence and relative risk of diabetes in people with various categories of dysglycemia: A systematic overview and meta-analysis of prospective studies. Diabetes Res. Clin. Pract..

[9] Tuomilehto J., Lindstrom J., Eriksson J.G., Valle T.T. (2001). Prevention of type 2 diabetes mellitus by changes in lifestyle among subjects with impaired glucose tolerance. N. Engl. J. Med..

[10] Diabetes Prevention Program Research Group (2002). Reduction in the incidence of type 2 diabetes with lifestyle intervention or metformin. N. Engl. J. Med..

[11] Aroda V.R., Christophi C.A., Edelstein S.L., Zhang P., Herman W.H., Barrett-Connor E., Delahanty L.M., Montez M.G., Ackermann R.T., Zhuo X. (2015). The effect of lifestyle intervention and metformin on preventing or delaying diabetes among women with and without gestational diabetes: The Diabetes Prevention Program outcomes study 10-year follow-up. J. Clin. Endocrinol. Metab..

[12] Cheung N.W., Smith B.J., Henriksen H., Tapsell L.C., McLean M., Bauman A. (2007). A group-based healthy lifestyle program for women with previous gestational diabetes. Diabetes Res. Clin. Pract..

[13] Kim C., Draska M., Hess M.L., Wilson E.J., Richardson C.R. (2012). A web-based pedometer programme in women with a recent history of gestational diabetes. Diabet. Med..

[14] Ferrara A., Hedderson M., Albright C., Ehrlich S., Quesenberry C., Peng T., Feng J., Ching J., Crites Y. (2011). A pregnancy and postpartum lifestyle intervention in women with gestational diabetes mellitus reduces diabetes risk factors: A feasibility randomized control trial. Diabetes Care.

[15] Goveia P., Cañon-Montañez W., De Paula Santos D., Lopes G.W., Ma R.C.W., Duncan B.B., Ziegelman P.K., Schmidt M.I. (2018). Lifestyle intervention for the prevention of diabetes in women with previous gestational diabetes mellitus: A systematic review and meta-analysis. Front. Endocrinol..

[16] Lim S., Dunbar J.A., Versace V.L., Janus E., Wildey C., Skinner T., O’Reilly S. (2017). Comparing a telephone- and a group-delivered diabetes prevention program: Characteristics of engaged and non-engaged postpartum mothers with a history of gestational diabetes. Diabetes Res. Clin. Pract..

[17] Lim S., Tan A., Madden S., Hill B. (2019). Health professionals’ and postpartum women’s perspectives on digital health interventions for lifestyle management in the postpartum period: A systematic review of qualitative studies. Front. Endocrinol..

[18] Nicklas J.M., Zera C.A., England L.J., Rosner B.A., Horton E., Levkoff S.E., Seely E.W. (2014). A web-based lifestyle intervention for women with recent gestational diabetes mellitus: A randomized controlled trial. Obs. Gynecol..

[19] Appel L.J., Clark J.M., Yeh H.-C., Wang N.-Y., Coughlin J.W., Daumit G., Miller E.R., Dalcin A., Jerome G.J., Geller S. (2011). Comparative effectiveness of weight-loss interventions in clinical practice. N. Engl. J. Med..

[20] Hurkmans E., Matthys C., Bogaerts A., Scheys L., Devloo K., Seghers J. (2018). Face-to-Face Versus Mobile Versus Blended Weight Loss Program: Randomized Clinical Trial. JMIR mHealth uHealth.

[21] International Association of Diabetes and Pregnancy Study Groups Consensus Panel (2010). International association of diabetes and pregnancy study groups recommendations on the diagnosis and classification of hyperglycemia in pregnancy. Diabetes Care.

[22] World Health Organization (WHO) (2013). Diagnostic Criteria and Classification of Hyperglycaemia First Detected in Pregnancy.

[23] Boedt T., Dancet E., Lie Fong S., Peeraer K., De Neubourg D., Pelckmans S., van de Vijver A., Seghers J., Van der Gucht K., Van Calster B. (2019). Effectiveness of a mobile preconception lifestyle programme in couples undergoing in vitro fertilisation (IVF): The protocol for the PreLiFe randomised controlled trial (PreLiFe-RCT). BMJ Open.

[24] Bogaerts A., Bijlholt M., Mertens L., Braeken M., Jacobs B., Vandenberghe B., Ameye L., Devlieger R. (2020). Development and Field Evaluation of the INTER-ACT App, a Pregnancy and Interpregnancy Coaching App to Reduce Maternal Overweight and Obesity: Mixed Methods Design. JMIR Form. Res..

[25] Vidgen H.A., Gallegos D. (2014). Defining food literacy and its components. Appetite.

[26] Benhalima K., Van Crombrugge P., Verhaeghe J., Vandeginste S., Verlaenen H., Vercammen C., Dufraimont E., De Block C., Jacquemyn Y., Mekahli F. (2014). The Belgian Diabetes in Pregnancy Study (BEDIP-N), a multi-centric prospective cohort study on screening for diabetes in pregnancy and gestational diabetes: Methodology and design. BMC Pregnancy Childbirth.

[27] Matthys C., Meulemans A. (2015). Development and validation of general FFQ for use in clinical practice. Nutr. Metab..

[28] Harrison C., Thompson R., Teede H., Lombard C. (2011). Measuring physical activity during pregnancy. Int. J. Behav. Nutr. Phys. Act..

[29] Dalfrà M.G., Nicolucci A., Bisson T., Bonsembiante B., Lapolla A. (2012). Quality of life in pregnancy and post-partum: A study in diabetic patients. Qual. Life Res..

[30] Petrou S., Morrell J., Spiby H. (2009). Assessing the empirical validity of alternative multi-attribute utility measures in the maternity context. Health Qual. Life Outcomes.

[31] Kim C., Mcewen L.N., Piette J.D., Goewey J., Ferrara A., Walker E.A. (2007). Risk Perception for Diabetes among Women with Histories of Gestational Diabetes Mellitus. Diabetes Care.

[32] Shigaki C., Kruse R.L., Mehr D., Sheldon K.M., Bin G., Moore C., Lemaster J. (2010). Motivation and diabetes self-management. Chronic Illn..

[33] Levesque C.S., Williams G.C., Elliot D., Pickering M.A., Bodenhamer B., Finley P.J. (2007). Validating the theoretical structure of the Treatment Self-Regulation Questionnaire (TSRQ) across three different health behaviors. Health Educ. Res..

[34] Marteau T.M., Bekker H. (1992). The development of a six-item short-form of the state scale of the Spielberger State—Trait Anxiety Inventory (STAI). Br. J. Clin. Psychol..

[35] Van der Bij A.K., de Weerd S., Cikot R.J.L.M., Steegers E.A.P., Braspenning J.C.C. (2003). Validation of the Dutch Short Form of the State Scale of the Spielberger State-Trait Anxiety Inventory: Considerations for Usage in Screening Outcomes. Community Genet..

[36] Eriksson M., Lindström B. (2007). Antonovsky’s sense of coherence scale and its relation with quality of life: A systematic review. J. Epidemiol. Community Health..

[37] American Diabetes Association (2016). 2. Classification and diagnosis of diabetes. Diabetes Care.

[38] Grundy S.M., Brewer H.B., Cleeman J.I., Smith S.C., Lenfant C. (2004). Definition of metabolic syndrome: Report of the National Heart, Lung, and Blood Institute/American Heart Association conference on scientific issues related to definition. Circulation.

[39] Matsuda M., DeFronzo R.A. (1999). Insulin sensitivity indices obtained from oral glucose tolerance testing: Comparison with the euglycemic insulin clamp. Diabetes Care.

[40] Matthews D.R., Hosker J.P., Rudenski A.S., Naylor B.A., Treacher D.F., Turner R.C. (1985). Homeostasis model assessment: Insulin resistance and β-cell function from fasting plasma glucose and insulin concentrations in man. Diabetologia.

[41] Kahn S.E. (2003). The relative contributions of insulin resistance and beta-cell dysfunction to the pathophysiology of Type 2 diabetes. Diabetologia.

[42] Retnakaran R., Qi Y., Goran M.I., Hamilton J.K. (2009). Evaluation of proposed oral disposition index measures in relation to the actual disposition index. Diabet. Med..

[43] Devlieger H., Martens G., Bekaert A., Eeckels R. (2000). Standaarden van geboortegewicht-voor-zwangerschapsduur voor de vlaamse boreling. Tijdschr Geneeskd.

[44] Benhalima K., Jegers K., Devlieger R., Verhaeghe J., Mathieu C. (2016). Glucose intolerance after a recent history of gestational diabetes based on the 2013 WHO criteria. PLoS ONE.

[45] Ciwit B.V. (2018). Castor Electronic Data Capture.

[46] Saaristo T., Moilanen L., Korpi-Hyövälti E., Vanhala M., Saltevo J., Niskanen L., Jokelainen J., Peltonen M., Oksa H., Tuomilheto J. (2010). Lifestyle intervention for prevention of type 2 diabetes in primary health care: One-year follow-up of the finnish national diabetes prevention program (FIN-D2D). Diabetes Care.

